# Analysis of risk factors and prognosis of 253 lymph node metastasis in colorectal cancer patients

**DOI:** 10.1186/s12893-021-01276-2

**Published:** 2021-06-04

**Authors:** Shidong Hu, Songyan Li, Da Teng, Yang Yan, Haiguan Lin, Boyan Liu, Zihe Gao, Shengyu Zhu, Yufeng Wang, Xiaohui Du

**Affiliations:** 1 Department of General Surgery, the First Medical Centre, Chinese PLA General Hospital, 28 Fuxing Road, Beijing, China; 2 Department of Hospital Management, the First Medical Centre, Chinese PLA General Hospital, Beijing, China

**Keywords:** Lymph node dissection, Molecular indexes, Immunohistochemistry

## Abstract

**Background:**

This study aimed to explore potential risk factors for 253 lymph node metastasis, and to identify the prognostic impact of 253 lymph node metastasis in colorectal cancer patients.

**Methods:**

A retrospective study was conducted of 391 colorectal cancer patients who underwent surgical treatments that included 253 lymph node dissection. Clinicopathological features, molecular indexes and 1-year overall survival rates were analyzed.

**Results:**

Univariate analyses revealed the following risk factors for 253 lymph node metastasis: high preoperative levels of CEA, large tumour max diameters, and numbers of harvested lymph nodes, presence of vessel carcinoma emboli, low level of MSH6 and MLH1 immunohistochemical staining intensity. Multivariate analysis showed that elevated MLH1 immunohistochemical staining intensity was an independent protective factor for 253 lymph node metastasis (OR: 0.969, 95% CI 0.945, 0.994, P = 0.015). A significant difference was found in 1-year overall survival rate between 253 lymph node-positive and lymph node-negative colorectal cancer patients (88.9% vs.75.0%, P < 0.001).

**Conclusions:**

253 lymph node-positive colorectal cancer patients had a worse prognosis than the 253 lymph node-negative patients. 253 lymph node dissection may improve the prognosis of colorectal cancer patients with high risk factors for 253 lymph node metastasis.

## Background

Colorectal cancer (CRC) counts as one of the most common malignancies. According to the latest WHO statistics, CRC has become the second most prevalent malignancy in women (after breast cancer) and the third most prevalent malignancy in men, with a total annual death toll of 861,700 worldwide [1]. Data released by the China National Cancer Centre show that colorectal morbidity and mortality rank fifth among all malignancies, and that they are increasing year after year, along with changes in lifestyle [2]. Colorectal cancer has become an important disease affecting public health in both Eastern and Western countries, and surgery is the most common approach to treat colorectal cancer.

The introduction of complete mesocolic-excision (CME) and total mesorectal-excision (TME) approaches, improvements in surgical instrumentation, as well as optimization of surgical techniques have enabled the standardization of colorectal cancer surgery and improved the efficacy of surgical treatment of patients with colorectal cancer [3, 4]. Lymph node metastasis is one of the prominent factors affecting the prognosis of patients with colorectal cancer, and the proper scope of intraoperative lymph node dissection remains controversial. Japanese researchers use 3 digits above 200 to indicate the large intestine lymph nodes. The 253 lymph nodes are regional lymph nodes at the roots of the inferior mesenteric artery (IMA), located between the beginning of the inferior mesenteric artery, the origin of the left colic artery (LCA) and the inferior mesenteric vein. They constitute the third station of lymphatic drainage for rectal cancer, with a metastasis rate of 0.3–11.1% [5–7]. Views on 253 lymph node dissection differ between Eastern and Western countries. Japanese surgical experts believe that 253 lymph node dissection is beneficial. The technique has become a standard operational method in Japan. Non-dissection of 253 lymph nodes is only used for patients whose tumours are confined to the muscle layer and in whom no lymph node metastasis has been found during preoperative examination [8]. In contrast, U.S. surgical experts recommend against the routine dissection of the 253 lymph nodes, and maintain that only when preoperative imaging confirms suspected metastasis among 253 lymph nodes, dissection should be considered [9].

How to identify the proper patients for 253 lymph node dissection, and whether 253 lymph node dissection promotes survival has thus far remained unresolved issues. In this study, we retrospectively analysed the clinicopathological characteristics of colorectal cancer patients who underwent 253 lymph node dissection at our medical centre, and conducted follow-up phone calls to develop selection criteria for the application of 253 lymph node dissection in colorectal cancer patients.

## Methods

### Study design and patients

**Fig. 1:**
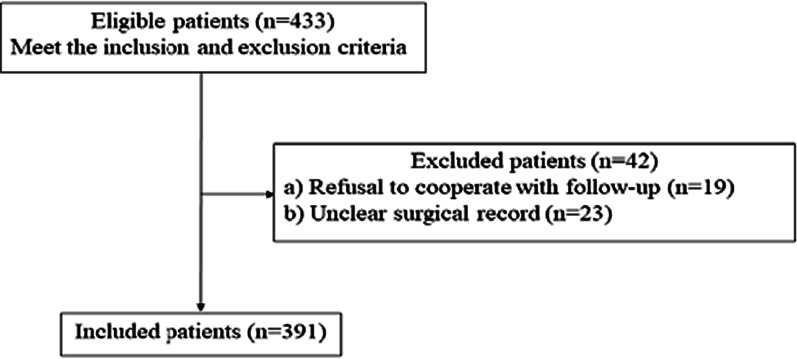
The flowchart of patients selection

We retrospectively selected 391 consecutive patients, aged 60.3 ± 10.8 year, with colorectal cancer, who underwent surgical treatment in our hospital between May 2015 and March 2020 and whose surgery included 253 lymph node dissection (Fig. 1). Among these patients, ten were 253 lymph node-positive. The inclusion criteria for this study were as follows: (1) aged 18 years or more; (2) confirmed malignancy in enteroscopic biopsies; (3) preoperative imaging indicating feasibility of radical surgical resection; (4) ASA classification score between I and III. Exclusion criteria included: (1) preoperative imaging suggesting enlarged IMA root lymph nodes; (2) absence of medical records of the patient. TNM staging was conducted as prescribed in the American Joint Committee on Cancer (AJCC) TNM Staging System for Colorectal Cancer (eighth edition, 2017). All contributing surgeons had associate-chief-physician or more senior positions at the hospital. Routine exploration of the abdominal cavity for distant metastasis, tumour location, penetration of the serous membrane, and complete colorectal cancer surgery were performed according to either CME or TME principles. The 253 lymph node dissection was performed within a triangular region, located between the left colonic artery/inferior mesenteric vein, around the inferior mesenteric artery, and the urogenital fascial surface at the root of the inferior mesenteric artery. The study was approved by the ethics committee of the Chinese PLA general hospital. Because of the retrospective nature of the research, the requirement for informed consent was waived by the ethics committee of the Chinese PLA general hospital. The study protocol is performed in accordance with the relevant guidelines.

### Data collection

The following baseline clinical variables were included in the data analysis: age at baseline, gender, location of the tumour, distance of the tumour from the anal verge (cm), distance between the lower margin of the tumour and the anal margin as determined by colonoscopy (cm), and preoperative levels of CEA/CA199/CA724. Routine postoperative pathological indicators included as variables were: differentiation, tumour max diameter, TNM class (i.e., T/N/M/TNM), histopathological type, presence of nerve invasion, presence of vessel carcinoma embolus, number of 253 lymph nodes, presence of cancerous nodes, number of harvested lymph nodes, number of metastatic lymph nodes. The molecular immunohistochemical indexes included those for HER1, HER2, KRAS, NRAS, BRAF, PIK3CA, Ki67, MLH1, MSH6, MSH2, PMS2 and mismatch repair (MMR) were examined by immunohistochemistry in the Department of Pathology. The Department of pathology performed a quantitative analysis of some positive immunohistochemistry indexes including Ki67, MLH1, MSH6, MSH2, PMS2.

Patient follow-up was conducted over the telephone, and the median follow-up period was 15 months. The follow-up was completed early May 2021. Overall survival (OS) was defined as the time from the completion of the surgery to the follow-up date, or date of death of the patient.

### Statistical analysis

Statistical analyses were performed with EmpowerStats (based on R language). All data distributions were evaluated using the Kolmogorov–Smirnov test to evaluate normality, and Levene’s test to evaluate the homogeneity of variance. Continuous variables are reported as median values and interquartile ranges (i.e., the difference between the 75th and 25th percentiles). Student’s t-tests were used to compare the statistical difference between the two patient groups, and one-way analysis of variance was used for comparison of multiple groups with additional Student–Newman–Keuls tests for pairwise comparisons). To analyse parameters that did not conform to normality or show homogeneity of variance, non-parametric Mann-Whitney U tests were used for comparison of statistical differences between the two patient groups, and Kruskal–Wallis rank tests were used to compare multiple groups. Chi-square tests were used to test statistical differences of counting data. Logistic regression was used for risk factor analysis. The Kaplan-Meier method was used for survival analysis, and log-rank tests were used for comparisons of differences. P-values of < 0.05 were considered to be statistically significant.

## Results

### Baseline clinical characteristics

This study included 391 patients with colorectal cancer who underwent complete intraoperative dissection of 253 lymph nodes, including 10 patients who also showed 253 lymph node metastases. No statistically significant differences in preoperative clinical baseline data between the two patient groups were found (Table 1).

**Table 1:** Patients baseline clinical characteristics

	253 lymph node negative group (n = 381)	253 lymph node positive group (n = 10)	P
Age: years	61.0(54.0–67.0)	59.5(43.5–70.0)	0.604
Gender			0.504
Male	135 (35.4%)	2 (20.0%)	
Female	246 (64.6%)	8 (80.0%)	
Preoperative level of CEA (ug/L)	3.1(1.9–7.3)	8.7 (3.2–17.6)	0.083
Preoperative level of CA199 (ug/L)	12.3 (6.8–21.4)	10.3 (7.2–21.8)	0.790
Preoperative level of CA724 (ug/L)	1.9 (1.2–3.9)	1.6 (1.3–12.5)	0.779
Distance of tumour from anal verge(cm)	10.0 (6.0–16.0)	16.5 (14.8–23.5)	0.066

### Postoperative pathological routine indicators

No statistically significant differences between the two groups were found for tumour location, T stage, nerve invasion and presence of cancerous nodes (Table 2). Tumour max diameter, number of harvested lymph nodes, number of metastatic lymph nodes, and number of 253 lymph nodes in the 253 metastatic patient group were all significantly higher than those in the non-metastatic group (5.0 + 2.0 vs. 3.8 + 1.8, P = 0.034; 18.0 + 7.1 vs. 14.4 + 4.9, P = 0.027; 0.0 vs. 4.0, P < 0.001; 1.0 vs. 2.0, P = 0.036) (Table 2). Statistical between-group differences were found in the differentiation, histopathological type, N stage, M stage, TNM stage, and vessel carcinoma embolus distribution (Table 2).

**Table 2:** Postoperative pathological routine indicators

	253 lymph node negative group (n = 381)	253 lymph node positive group (n = 10)	P
Tumor location			0.087
Rectum	254 (66.6%)	3 (30.0%)	
Sigmoid colon	94 (24.7%)	6 (60.0%)	
Rectum sigmoid colon junction	9 (2.4%)	1 (10.0%)	
Others	24 (6.3%)	0 (0.0%)	
Tumor max diameter: cm	3.8 ± 1.8	5.0 ± 2.0	0.034
Differentiation			0.011
Well	3 (0.9%)	0 (0.0%)	
Moderately	284 (83.8%)	4 (44.4%)	
Poorly	52 (15.3%)	5 (55.6%)	
Histopathological type			0.016
Adenocarcinoma	329 (91.9%)	8 (80.0%)	
Mucinous adenocarcinoma	11 (3.1%)	0 (0.0%)	
Signet-ring cell carcinoma	3 (0.8%)	1 (10.0%)	
Others	15(4.2%)	1 (10.0%)	
T stage			0.118
Tis	2 (0.6%)	0 (0.0%)	
T1	21 (5.8%)	0 (0.0%)	
T2	57 (15.8%)	1 (10.0%)	
T3	230 (63.7%)	6 (60.0%)	
T4a	48 (13.3%)	2 (20.0%)	
T4b	3 (0.8%)	1 (10.0%)	
N stage			< 0.001
N0	230 (61.5%)	0 (0.0%)	
N1a	47 (12.6%)	2 (20.0%)	
N1b	43 (11.5%)	1 (10.0%)	
N2a	29 (7.8%)	3 (30.0%)	
N2b	25 (6.7%)	4 (40.0%)	
M Stage			< 0.001
M0	367 (98.1%)	7 (77.8%)	
M1	6 (1.6%)	1 (11.1%)	
M1b	1 (0.3%)	0 (0.0%)	
M1c	0 (0.0%)	1 (11.1%)	
TNM stage			0.049
I	23 (6.4%)	0 (0.0%)	
II	130 (36.1%)	0 (0.0%)	
III	143 (39.7%)	6 (60.0%)	
IV	64 (17.8%)	4 (40.0%)	
Nerve invasion			0.209
No	342 (91.4%)	8 (80.0%)	
Yes	32 (8.6%)	2 (20.0%)	
Vessel carcinoma embolus			0.001
No	338 (90.9%)	6 (60.0%)	
Yes	34 (9.1%)	4 (40.0%)	
Cancerous node			0.134
No	323 (86.6%)	7 (70.0%)	
Yes	50 (13.4%)	3 (30.0%)	
Number of harvested lymph nodes	14.4 ± 4.9	18.0 ± 7.1	0.027
Number of metastatic lymph nodes	0.0 (0.0–2.0)	4.0 (2.5–7.8)	< 0.001
Number of 253 lymph nodes	1.0 (0.0–3.0)	2.0 (1.2-4.0)	0.036

### Molecular indexes

The immunohistochemical intensities of MSH6 and MLH1 in the 253 lymph-node metastasis patient group were significantly higher than those in patients without lymph-node metastases (78.9% + 13.1% vs. 68.0% + 26.1%, P = 0.013; 72.5% + 15.6% vs. 56.5% + 24.3%, P = 0.002). The other molecular immunohistochemical indexes that were analysed (i.e., Ki67, MSH2, PMS2, MMR, HER1, HER2, KRAS, NRAS, BRAF and PIK3CA) showed no statistical differences (Table 3).

**Table 3:** Molecular immunohistochemical indexs

	253 lymph node negative group (n = 381)	253 lymph node positive group (n = 10)	P
Ki67	74.5% ± 15.6 %	79.0% ± 8.8 %	0.371
MSH2	79.4% ± 12.7 %	75.5 ± 11.7	0.340
MSH6	78.9% ± 13.1 %	68.0% ± 26.1 %	0.013
MLH1	72.5% ± 15.6 %	56.5% ± 24.3 %	0.002
PMS2	70.9% ± 17.8 %	60.0% ± 21.2 %	0.059
MMR			0.602
DMMR	9 (2.7%)	0 (0.0%)	
PMMR	330 (97.3%)	10 (100.0%)	
HER1			0.813
Negative	42 (12.5%)	1 (10.0%)	
Positive	294 (87.5%)	9 (90.0%)	
HER2			0.647
Negative	89 (27.1%)	3 (30.0%)	
+	157 (47.7%)	3 (30.0%)	
++	81 (24.6%)	4 (40.0%)	
+++	2 (0.6%)	0 (0.0%)	
KRAS			0.965
Wild type	72 (65.5%)	2 (66.7%)	
Mutant type	38 (34.5%)	1 (33.3%)	
NRAS			0.678
Wild type	104 (94.5%)	3 (100.0%)	
Mutant type	6 (5.5%)	0 (0.0%)	
BRAF			0.869
Wild type	110 (99.1%)	3 (100.0%)	
Mutant type	1 (0.9%)	0 (0.0%)	
PIK3CA			0.813
Wild type	107 (98.2%)	3 (100.0%)	
Mutant type	2 (1.8%)	0 (0.0%)	

### Risk factors for 253 lymph node-positive metastasis

Univariate analysis of the risk factors for 253 lymph node metastasis showed that CEA, tumour max diameter, presence of vessel carcinoma embolus, number of harvested lymph nodes and MSH6- and MLH1-expression levels were associated with 253 lymph-node metastasis (Table 4). High expression of MLH1, as shown by immunohistochemistry was an independent protective factor for 253 lymph-node metastases (OR: 0.969, 95% CI 0.945, 0.994, P = 0.015) (Table 4).

**Table 4:** Risk factors for 253 lymph node positive

	Univariate analysis		Multivariate analysis	
	P	Odds ratio (95% CI)	P	Odds ratio (95% CI)
CEA	0.052	1.0 (1.0, 1.0)	> 0.05	
Tumor max diameter: cm	0.034	1.4 (1.0, 2.0)	> 0.05	
Vessel carcinoma embolus	0.005	6.6 (1.8, 24.6)	> 0.05	
Number of harvested lymph nodes	0.029	1.1 (1.0, 1.2)	> 0.05	
MSH6	0.023	0.971 (0.946, 0.996)	> 0.05	
MLH1^a^	0.005	0.966 (0.943, 0.989)	0.015	0.969 (0.945, 0.994)

^a^MLH1 Multivariate analysis Adjust model adjust for:MSH6

### Long-term outcomes

**Fig. 2:**
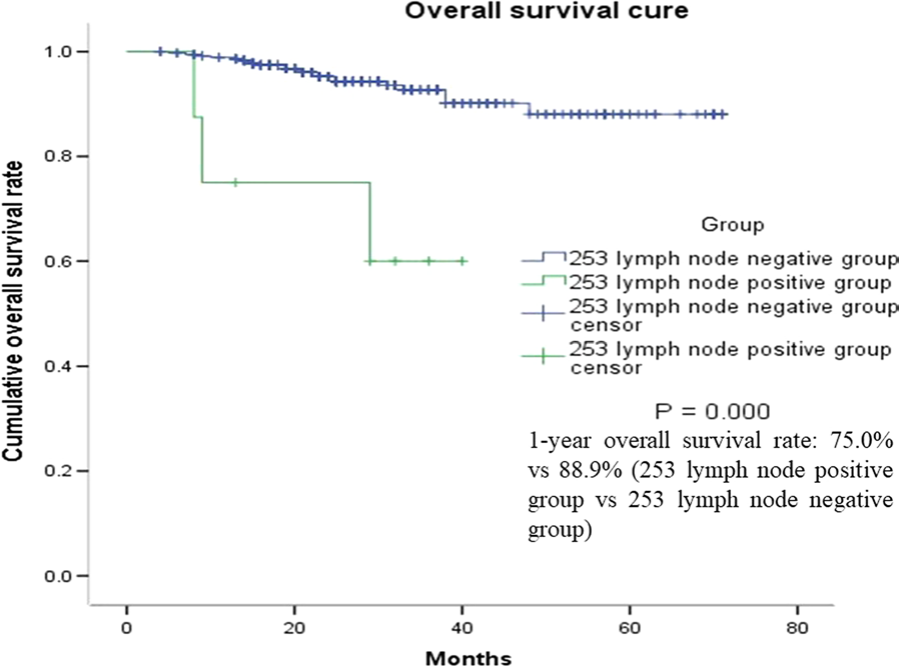
Overall survival cure of different groups

There was a significant difference in 1-year overall survival rate between patients with 253 lymph-node metastasis and those without metastasis (88.9% vs.75.0%, P < 0.001) (Table 5; Fig. 2).

**Table 5:** Long-term outcomes

	253 lymph node negative group (n = 381)	253 lymph node positive group (n = 10)	P
1-year overall survival rate	88.9 %	75.0 %	0.000

## Discussion

The most common metastatic route of colorectal cancer is via the lymphatic system. The occurrence of 253 lymph node-metastases among colorectal cancer patients reported in the literature is low, between 0.3 and 11.1% [5–7]. Our study found an overall rate of metastasis in 253 lymph nodes of 2.6%, based on specific rates in patients with pT1, pT2, pT3, and pT4 colorectal cancer of 0%, 1.7%, 2.5%, and 5.6%, respectively. A Japanese study found that the rates of 253 lymph node metastasis in pT1, pT2, pT3 and pT4 were 1%, 1%, 2.7 and 10%, respectively [10], which is similar to our findings. The risk factors of 253 lymph-node metastasis were important for colorectal cancer patients.

Our univariate analysis found some potential risk factors which should be confirmed in future studies. Clinical pathological risk factors for 253 lymph node metastasis include preoperative CEA levels, tumour max diameter, the presence of vessel carcinoma embolus, and the number of harvested lymph nodes. Molecular-pathological analysis indicated that low expressions of MSH6 and MLH1 as measured by immunohistochemistry were potential risk factors for 253 lymph node metastasis. The potential metastatic risk factors we identified have also been reported in previous studies. CEA is a tumour-associated antigen that has been detected in colon-cancer and embryonic tissues. which is widely used as a marker in clinical screening of gastrointestinal tumours [11]. Prior studies found that preoperative CEA levels are positively correlated with 253 lymph-node metastasis [12–14], a finding which was confirmed by our study. The median preoperative CEA levels of patients with 253 lymph-node metastasis were significantly higher than those of patients without 253 lymph-node metastasis (8.7 vs. 3.1). Due to the limited sample size for patients with this type of metastasis, the observed difference failed to attain statistical significance. Previous studies showed that tumour size is associated with the occurrence of 253 lymph-node metastasis [15]. Our results showed that patients with 253 lymph-node metastasis had higher tumour max diameters than patients without metastasis (5.0 + 2.0 vs. 3.8 + 1.8, respectively; P = 0.034). Univariate regression analysis found that the tumour max diameter was a risk factor for the occurrence of 253 lymph node metastasis (OR = 1.4, 95% CI 1.0–2.0, P = 0.034). A tumour with strong growth ability may also have strong lymph node metastasis ability. Vessel carcinoma embolus refers to tumour invasion of peripheral venules or lymphatic vessels. Evidence shows that vascular carcinoma thrombus is associated with 253 lymph node metastasis [12]. We found that the proportion of vessel carcinoma embolus in 253 lymph node metastasis patients was significantly higher than in non-metastatic patients (60% vs. 90.9%, P = 0.001), and the result of univariate regression analysis was OR = 6.6 (95 %CI 1.8–24.6, P = 0.005). The number of harvested lymph nodes can reflect the quality of surgical resection. An earlier study showed that the number of harvested lymph nodes had no significant correlation with 253 lymph node metastasis [12]. We found that 253 lymph node metastasis patients harvested more lymph nodes, however multivariate regression analysis did not find a correlation between harvested lymph nodes and 253 lymph node metastases. Microsatellite instability (MSI) is associated with the failure of one or more MMR proteins, which is the primary cause of Lynch syndrome [16]. It was found that compared with microsatellite stability (MSS), MSI was less likely to lead to lymph node metastasis [17]. Immunohistochemistry was used to detect the expression of tumour tissue mismatch repair proteins MLH1, MSH2, MSH6, and PMS2, which could reflect the MSI status. MLH1, MSH2, MSH6, and PMS2 were all expressed as PMMR, otherwise DMMR. Our study found that MMR status was not related to 253 lymph node metastasis, however, the immunohistochemical staining intensities of MSH6 and MLH1 in the 253 lymph node metastasis group were weaker than those in the non-metastatic group (68.0 ± 26.1% vs. 78.9 ± 13.1%, P = 0.013; 56.5 ± 24.3% vs. 72.5 ± 15.6%, P = 0.002). Multivariate logistic regression analysis found that the increased immunohistochemical staining intensity of MLH1 was a protective factor in non-metastatic patients (OR: 0.969, 95 %CI 0.945–0.994, P = 0.015), and the loss of MLH1 expression might lead to 253 lymph node metastasis. There was no statistical difference in Ki67, HER1, HER2 between the 253 lymph node metastatic and the non-metastatic groups. However, we cannot confirm that there is no statistically significant difference in RAS gene phenotype, BRAF gene phenotype and PIK3CA gene phenotype between the 253 lymph node metastatic and the non-metastatic groups due to a small number of patients presented the values of KRAS, NRAS, BRAF, PIK3CA.

253 lymph node metastasis was associated with the prognosis of colorectal cancer patients. The present study found that patients with 253 lymph node metastasis often had other regional lymph node metastases, however, the 253 lymph nodes had leaping metastasis. In 6% of patients, the 253 lymph nodes were the only lymph node that is affected. The 253 lymph nodes were of great significance for prognosis evaluation and lymphatic staging of the patients [18, 19]. The 5-year survival rates of patients with positive and negative 253 lymph nodes were 31 and 50%, respectively (P = 0.004). 253 lymph node metastasis was an independent risk factor affecting the long-term prognosis [18]. The study found that the 5-year survival rate of colorectal cancer patients with 253 lymph node metastasis was similar to that of those with distant metastasis. Even though the 253 lymph node was the only metastatic lymph node, the prognosis was still poor [20]. Our study found that the 1-year OS of patients with 253 lymph node metastasis was significantly lower than that in patients without 253 lymph node metastasis (75.0% vs. 98.9%, P < 0.05). Studies on the potential value of 253 lymph node dissection for improving the prognosis of patients with 253 lymph node metastasis are limited. A meta-analysis involving 3119 patients showed that dissection of 253 lymph nodes can improve 5-year survival (HR: 0.77, 95% CI 0.66–0.89) [21]. Whether 253 lymph node dissection can improve the prognosis of colorectal cancer patients without 253 lymph node metastasis has not been determined. The results of multiple clinical studies have not confirmed that 253 lymph node dissection can improve the prognosis [22–25]. Hiroaki Inoue et al. found that 5-year OS was significantly different between patients without and with 253 lymph node metastasis (83.3% vs. 36.2%, P < 0.0001) and the therapeutic value index of 253 lymph node was 0.54 [26]. Y Kanemitsu et al. found 1.7% had 253 lymph nodes and 99 (8.3 per cent) had metastases to station 252. The 5- and 10-year survival rates of patients with 253 lymph node metastasis were 40 and 21% respectively [27]. Most clinical studies that examined the role of 253 lymph node dissection in the prognostic value of colorectal cancer did not yield positive results, which may be related to the low rate of 253 lymph node metastasis in colorectal cancer patients. Assessment of preoperative indicators, including colonoscopic biopsies for molecular pathological detection, precise imaging assessment of tumour staging and other methods were used to screen high-risk factors for 253 lymph node metastasis.253 lymph node dissection may improve the prognosis of high-risk populations.

## Limitations

A limitation of our study was that it was single-centre, and retrospective. The examined number of 253 lymph node metastasis patients was small, and the results were biased. More conclusive evidence regarding the possible benefits of 253 lymph node dissection to reduce metastatic risk should be provided by future, prospective multicentre studies.

## Conclusions

In this study, we examined potential risk factors for 253 lymph node metastasis in colorectal cancer patients, and found increased metastatic risk in patients with high preoperative levels of CEA, large tumour max diameters, the presence of vessel carcinoma or emboli, higher numbers of harvested lymph nodes, and reduced expression of MSH6 and MLH1 as detected by immunohistochemistry. The prognoses of colorectal cancer patients with 253 lymph node metastasis were worse than those in patients without 253 lymph node metastasis. Thus, 253 lymph node dissection may improve the prognosis of colorectal cancer patients with a high risk of 253 lymph node metastasis.

## Data Availability

The datasets used and/or analysed during the current study are available from the corresponding author on reasonable request.

## Abbreviations

CRC: Colorectal cancer
CME: Complete mesocolic-excision
TME: Total mesorectal-excision
IMA: Inferior mesenteric artery
LCA: Left colic artery
MMR: Mismatch repair
OS: Overall survival
MSI: Microsatellite instability
MSS: Microsatellite stability
